# Transactions of the Buffalo Obstetrical Society

**Published:** 1885-04

**Authors:** 


### TRANSACTIONS OF THE BUFFALO OBSTETRICAL SOCIETY.

*Stated Meeting, Feb. 23, 1885.*

The Vice-President, Dr. R. L. Banta, in the Chair; Dr. William H. Thornton, Secretary.

Dr. W. W. Potter read a paper on "Pessaries; their Use and Misuse."

This subject, he said, always brought forth a diversity of opinion as to the real therapeutic value of pessaries, ranging all the way from cordial approval of their use to hostile criticism thereof. The reasons therefor seemed to be—

1. In the wide difference in the degree of displacements; in the multiform reflex disturbances which they provoke; in adjacent surrounding and modifying conditions; in sensitiveness of the pelvic tissues; and in a variety of peculiarities associated with, and underlying, the mere uterine abnormalities.

2. In the different views held by physicians as to the mechanics of the treatment.

3. In the inability of anatomists to fix the precise normal position of the womb. The province of the gynecologist was, he thought, fulfilled if he succeeded in restoring the uterus to that approximate degree of normality which relieved the symptoms caused by the mal-position of the organ.

He considered it essential to replace the dislocated organ, or organs, before any attempt was made to insert a pessary. If it appeared, in any case, impossible to do this by reason of the stability, impaction or fixation of the uterus, or its appendages, then it was not a proper one for a pessary; not, certainly, until these contra-indicating factors were removed by a carefully systematized preparatory treatment. If the broad ligaments were tender, if the ovaries were enlarged or swollen, or tender, if the tubes were inflamed or diseased in any way, or if the womb itself were hypersensitive, enlarged, hyperplastic or impacted, it were better, before attempting the employment of a pessary, to begin the treatment with the application, to the vagina and pelvic organs, of cotton pledgets soaked in alum-glycerine or other tonic-astringent medicine. Iodoform, iodine, tannin, carbolic acid, sedative mixtures, eucalyptus, and even vasaline might also be employed with benefit in preparing cases for the use of pessaries. This method of vaginal packing served a three-fold purpose—

1. It soothed and kept asunder the irritated and hypersensitive tissues, acted something like a poultice to the parts, and set up capillary drainage by osmosis, thereby relieving congestion, hyperæmia and pelvic blood-stasis.

2. It made equable elastic pressure upon the enlarged, indurated, or swollen structures with which it came in contact, thus promoting absorption and resolution.

3. It furnished a comfortable and much needed support to the sagging and descending pelvic organs, removed the tension from the uterine ligaments and prevented their further stretching and elongation. Furthermore, if there were adhering bands of plastic lymph, or other products of cellulitis, which moored the uterus in the sacral excavation, these usually stretched out, absorbed, or gave way under this plan, so that the organs could be made to float in the pelvic cavity.

The reposition of the womb could now be undertaken, and he usually employed the knee-chest posture for this purpose, thereby dispensing with the sound or other repositor. In this position the abdominal and pelvic organs gravitated towards the epigastrium, the air filled the vagina with a *vis a tergo* pressure of fifteen pounds to the square inch, and the uterus, its appendages, and other pelvic organs, were carried to their proper level. The application of a pessary could now be made in full view of the parts, and with less danger of failure than the adjustment made in the dorsal position.

He then gave explicit directions as to the application of the instrument and its future care while in use, laying much stress upon the importance of the frequent employment of hot vaginal lavements by the patient during the progress of the treatment, and insisted upon the paramount necessity of her reporting herself to her physician at such intervals as he should direct, that he might not only satisfy himself that the pessary was doing no harm, but that he might also remove it sufficiently often to ensure its cleanliness and general efficiency, as well as discontinue its use when its purpose had been served. A patient must be instructed in such a way that she will understand she is regarded as under treatment just so long as she wears a pessary. The ill-repute of treatment by pessaries originated, not so much from the improper or faulty adjustment in the first instance, as from imprudence or neglect in their care afterwards. It was not uncommon for women to wear pessaries for months, and even years, without having them removed or otherwise cared for by their medical advisers. They seemed to have the impression that, with the first successful reposition of the womb and adjustment of a pessary, all medical care or treatment for the difficulty in question was at an end. No more dangerous impression could obtain, so far as the successful employment of pessaries was concerned. It was their misuse or abuse which brought discredit to the treatment. If neglected, they dig sulci in the soft parts, become imbedded in the tissues, or incarcerated by adventitious bands, so that surgical means were often required for their liberation. He related a case which he had lately seen, where a Babcock's uterine supporter had cut a fistulous opening into the bladder at the cervico-vaginal junction, an inch in length, from which there was a constant dribbling of urine. He considered Babcock's supporter a most unscientific appliance, an abomination of gynecological art; it was not, after all, a matter of such great surprise that women should become prejudiced against the art when such abominations were practiced upon them in its name.

He next referred to the condition of the perineum, which exerted a marked influence over the conduct of a pessary. If there was a considerable rent thereof, it must be repaired before much good could be obtained from a pessary. All such make-shifts as globe, ring, or disk pessaries, in these cases, would not avail; perineorrhaphy must be made. He pointed out the benefit to be obtained from a pessary in the retroverted or flexed gravid womb; uncontrollable pregnancy vomiting might be relieved, and threatened abortion averted, by the timely reposition of the organ and the judicious use of mechanical support.

He regarded the treatment of every variety of uterine displacement as a problem in mechanics, and no one should undertake it unless he had a thorough conception of the mechanical questions involved, in both diagnosis and treatment. Here the principles of orthopedic surgery were to be applied, and when we could bring ourselves to regard a pessary as a crutch or splint, for a weakened, heavy, or distorted womb, with a relaxed state of its ligaments and other supporting tissues, we would be traveling far in the right direction. It would be more satisfactory if the pessary were removed at night and replaced in the morning; only in exceptional cases, however, could patients do this. Nevertheless, we could do something akin to it, which answered nearly as well, by instructing patients who have worn pessaries during the day to loosen them up at night by getting on their knees and breast and admitting the air into the vagina by separation of the labia. This would practically unship the instrument from its bearings, take off the weight of the superincumbent viscera, and rest the parts amazingly. With the resumption of the erect posture in the morning the crutch-like action of the pessary was also restored, and it was worn all day without unduly chafing the tissues, for they came to their work restored and with renewed vigor.

*Discussion*—Dr. W. S. Tremaine said that, from his point of view, a pessary is always to be regarded as a necessary evil. It was well compared to a splint in case of a fracture, should only be used in the same sense, and never used when it could be avoided. He would like to know whether any better pessary than the Hodge, with its various modifications, has ever been devised? It seemed to him that it had never been, as a principle, much improved upon. In regard to the rectification of malpositions of the uterus, he had given some attention to Alexander's operation, for the relief of prolapsus, by shortening the round ligaments. Though he had never made the operation on a living woman, he had done it several times upon the cadaver. He thought the operation worthy of attention, and believed it had a future. In regard to rectifying displacements, he thought the treatment by tamponade, as advocated, a useful method, and spoke of a way of using lamp wicking for this purpose, which Dr. Foster, of New York, taught him some years ago; by smearing it with iodoform ointment it could be made antiseptic, and it could be easily removed by the patient.

Dr. Thomas Lothrop wished to speak in special commendation of that portion of the paper which dealt with the employment of the knee-chest posture, in rectifying mal-positions of the uterus and other organs. He believed it possessed special value in these cases, and had seen great good result from the use of the method. He thought the problems involved in the subject of uterine displacements, and their treatment by pessaries, very difficult ones to grapple with, in many instances, and coincided with the principles advocated in the paper in their management.

Dr. W. D. Greene, referring to what had been said in regard to the length of time pessaries may remain in *situ*, recalled a case which came to his notice about six years ago, where he removed a pessary that had been worn twelve years without removal. It was covered with the salts of the discharges, which had crystalized on the pessary, yet he was surprised to find that it had caused no very extensive abrasions. It was a gutta-percha pessary.

Dr. S. Y. Howell thought that the use of the ring pessary, in view of its simplicity, ought to be more generally resorted to. He advocated its use for ante-, retro- and lateral displacements of the uterus, stating that he found it extremely practical, and easily adaptable to individual cases. He was cognizant of cases where they had been worn for months without attention, and apparently without causing any ill effects.

Dr. M. Hartwig related an interesting case of total prolapsus uteri, which he replaced and held in position with a Meyer's ring, which is a very thick hard rubber instrument. The consequence was, violent fever of an intermittent type. They (another physician consulting with him) gave quinine, thinking it a mere coincidence. It did no good. The woman went to bed and they removed the pessary, whereupon the fever subsided. After awhile he introduced the pessary, and the fever started again. He thought the malaria had not died out and gave more quinine. She had to lie in bed, and, for convenience, he took out the pessary while she was in bed. The fever at once subsided and he introduced the pessary again with the same results as on the previous trials. He then thought it must be due to the absorption of putrescent material, and took extreme precautions against such a possibility at the next application of the pessary; but the fever appeared the fourth time. He, thereupon, abandoned the use of the pessary altogether, and the fever then abandoned the patient. In cases of total prolapsus he thought a simple round pessary served the purpose better than any other, and spoke of the especial merits of the Meyer's ring in this class of cases.

Dr. H. D. Ingraham was a firm believer in the usefulness of pessaries, and thought, in safe hands, they could be made to do a vast amount of good. It seemed to require an especial tact, however, to succeed with them in many cases, for a considerable proportion of disorders were of a nature to tax the patience, skill and ingenuity of the gynecologist to the uttermost. He was more successful with the Hodge pessary, including its modifications, than any other.

Dr. R. L. Banta had listened to the paper with the greatest interest, for he had witnessed many of the manipulations and methods described in the paper, having seen Dr. Potter successfully apply the principles which he advocated, in both dispensary and private practice. In retroversion, where a pessary can be borne at all, he thought almost any modification of the Hodge pessary would yield good results, but he particularly favored the Albert Smith form. He had seen remarkable results from the tamponades, where the womb was bound down, and where there was great danger to the patient in putting it back. He remembered that some years ago a lady came to him with a retroverted womb bound down by adhesions. He was not able to do anything with the case, and took her to another physician in this city, who also thought that nothing could be done for her. He had seen just such cases successfully treated by the essayist, according to the methods which he had set forth in the paper.

Dr. Potter, in closing the discussion, remarked that he had sought to elaborate principles only in his paper, as its title would indicate, rather than to discuss the special application of pessaries to particular displacements. Failures in the use of pessaries, he thought, resulted principally from the fact that their employment was begun before cases were properly prepared for them, hence they were not tolerated. The Hodge pessary, no doubt, represented the ideal principle nearer than any other, and he especially commended the Albert Smith and Thomas modifications. With regard to the ring pessary, he had, in former years, used them quite extensively, but he regarded them as unsurgical devices, for they distended the vagina too much. They were, however, possessed of a limited adaptability, notably for such cases as described by Dr. Hartwig. He, however, used the copper wire ring covered with soft rubber, as a model in some cases where special shapes were required, fashioning such a design as would suit the case, and then would have a hard rubber pessary constructed according to the model.

Concluding, he grouped certain principles which were involved in the use of pessaries as follows:

1. A patient should be properly prepared for the wearing of a pessary before its adjustment was attempted. Vaginal irritation, hyperæsthesia, fixation of the uterus, ovarian prolapse, salpingitis, and other contra-indicating conditions may generally be removed by a systematic medicated tamponade of the vagina, continued for a longer or shorter period.

2. It was essential to replace the womb before attempting to adjust a pessary. To neglect this was as unscientific as it was unsurgical to dress a fracture before reducing it. We could not know how to adjust the mechanical support required, until after full replacement of the womb.

3. The fullest replacement of the womb could only be made in the knee-chest posture.

4. A pessary was always to be adjusted with due and ample regard for the safety of the soft parts. Should it fit too tightly, or fill the vagina so closely that there was no play between its bearings and the vaginal wall, erosion or ulceration would quickly result. Neither should it be large enough to cause uterine fixation—the normal mobility of the organ and its respiratory oscillation should not be abridged. On the other hand, a pessary should not be small enough to cause erosions, nor to get out of place easily. Great practice was required for the acquisition of a skilled touch in the matter of a safe and proper fitting of the instrument.

5. A pessary should be adjusted with regard to the functions of the organs, vessels, and nerves of the pelvis, that they may not be interfered with nor impaired in any way.

6. In a well-adjusted instrument, the patient would not be aware of its presence from any sensations which it produced; if she were so reminded of it, the almost certain inference would be that it was illy adapted to her case.

7. Finally, every woman who wore a pessary should be instructed to return, at appropriate intervals, for its inspection, and made to understand that she is a patient under observation as long as she requires the support afforded by the instrument. Furthermore, she should be instructed to assume the genu-pectoral posture nightly on retiring, and admit air into the vagina, so as to unship or loosen up the bearings of the pessary, to thereby rest the parts, and the better prepare them for the continued tolerance of the instrument.

